# Effect and mechanism of vitamin D activation disorder on liver fibrosis in biliary atresia

**DOI:** 10.1038/s41598-021-99158-3

**Published:** 2021-10-06

**Authors:** Song Sun, Menghua Xu, Peijun Zhuang, Gong Chen, Kuiran Dong, Rui Dong, Shan Zheng

**Affiliations:** 1 Surgical Department, Children’s Hospital of Fudan University, 399 Wanyuan Road, Shanghai, 201102 China; 2 The Center of Laboratory Medicine, Children’s Hospital of Fudan University, Shanghai, 201102 China; 3 Anesthesiology Department, Children’s Hospital of Fudan University, Shanghai, 201102 China

**Keywords:** Nutrition disorders, Epidemiology, Paediatric research

## Abstract

To investigate the mechanism of 25 hydroxyvitamin D (25(OH)D) deficiency in children with biliary atresia (BA) and its effect on liver fibrosis. The serum vitamin D and 25(OH)D, and expression of 25 hydroxylase (CYP2R1 and CYP27A1) in the liver of BA patients were detected and compared with those in the control group. We investigated the effect of differential expression of CYP2R1 in hepatocytes on the expression of genes related to liver fibrosis in primary hepatic stellate cells (HSCs) of BA and animal models of cholestasis. The ratio of 25(OH)D/vitamin D in the BA group was significantly lower than that in the control group. The mRNA and protein expression of CYP2R1 and CYP27A1 in liver tissue of the BA group was significantly lower than that in the control group. Exogenous active vitamin D (calcitriol) inhibited the proliferation and migration of primary HSCs isolated from BA patients, and reduced the expression of fibrosis-related genes in vitro. Downregulation of expression of CYP2R1 in hepatocytes increased expression of transforming growth factor (TGF)-β1, collagen (Col)-1α1 and tissue inhibitor of metalloproteinase (TIMP)-1, and decreased the expression of matrix metalloproteinase (MMP)-2 in cocultured primary HSCs of BA. Upregulation of expression of CYP2R1 in mice with bile duct ligation significantly increased the level of 25(OH)D, decreased the expression of TGF-β1, Col-1α1 and TIMP-1, and increased the expression of MMP-2. Children with BA have impaired vitamin D activation due to CYP2R1 deficiency. The dysactivation of vitamin D can promote the proliferation and activation of HSCs and participate in the development of hepatic fibrosis in BA.

## Introduction

Biliary atresia (BA) is the most common biliary obstructive disease and the most common cause of jaundice in infants^1,2^. Hepatic portoenterostomy (HPE) before 90 days of age is the standard treatment for BA. Bile drainage was successfully established in 60–80% of the children with BA treated by HPE^1^; jaundice gradually decreased within 6 months, and liver function gradually tended towards normal. However, the majority of these children still had progressive liver fibrosis, which eventually progresses to cirrhosis, gastrointestinal bleeding and liver failure within a few years^3^. Therefore, exploration of the molecular biological mechanism and influencing factors of progressive liver fibrosis in BA is the basis for further improvement of its clinical prognosis. Vitamin D deficiency is widespread and severe in children with BA, and the association between vitamin D and liver fibrosis in chronic liver disease has been confirmed in recent years. The present study aimed to explore the intrinsic molecular biological mechanisms of vitamin D activation disorder and liver fibrosis in children with BA, so as to provide a new theoretical basis and therapeutic target for the antifibrosis treatment of BA.

## Materials and methods

### Overall study process

Ten children with BA were enrolled and 10 age-matched children (younger than 120 days of age) with choledochal cyst were selected as the control group. Clinically discarded blood samples from routine preoperative blood tests and intraoperative liver biopsy specimens from HPE procedures were collected. Serum vitamin D was detected by ELISA; serum 25 hydroxyvitamin D (25(OH)D) level was detected by electrochemiluminescence; mRNA expression of CYP2R1,CYP27A1 and vitamin D receptor (VDR) in liver tissue was detected by quantitative real-time polymerase chain reaction (qPCR); and protein expression of CYP2R1 and CYP27A1 was detected by immunohistochemistry and western blotting. The above indexes were compared between the BA and control groups.

Primary hepatic stellate cells (HSCs) of children with BA (n = 10) were isolated by density gradient centrifugation and verified through the expression of α-smooth muscle actin (SMA) detected by immunofluorescence assays. The effect of exogenous active vitamin D, calcitriol (1,25 dihydroxyvitamin D, 1,25(OH)_2_D) on the expression of fibrosis-related genes such as transforming growth factor (TGF)-β1, collagen (Col)-1α1 and α-SMA in primary HSCs was detected. The effect of calcitriol on the proliferation and migration of primary HSCs was tested by the CCK-8 assay and Transwell cell migration chambers. CYP2R1 overexpression lentivirus, CYP2R1 interference lentivirus, and negative control lentivirus were constructed by Shanghai Nuobai Biological Technology Co., Ltd. The lentiviruses were respectively transfected into QSG-7701 hepatic cells, and the coculture system of QSG-7701 and primary HSCs of BA was established. The effect of differential expression of CYP2R1 in hepatic cells on the expression of hepatic fibrosis-related genes in primary HSCs was observed.

CYP2R1 overexpression adeno-associated viruses (AAVs), CYP2R1 interference AAVs, and negative control AAVs were constructed by Shanghai Nuobai Biological Technology Co., Ltd. The common bile duct in C57BL/6 mice (n = 16) was ligated through laparotomy operation under general anesthesia by intraperitoneal injection of chloral hydrate. CYP2R1-overexpression AAV (1 × 10^12^ VP/ml, 100 μl), CYP2R1-interference AAV (1 × 10^12^ VP/ml, 100 μl), negative control AAV (1 × 10^12^ VP/ml, 100 μl) or 2 μg/kg calcitriol (positive control) was injected on the first day after surgery (4 mice per group). After 2 weeks of feeding, the mice were sacrificed by cervical dislocation, and the liver morphology and fibrosis of mice with biliary ligation were compared; in addition, the expression of genes related to liver fibrosis was tested by qPCR and western blotting and compared between the groups.

### ELISA and electrochemiluminescence

Serum vitamin D concentrations in the BA group (n = 10) and control group (n = 10) were tested with a human vitamin D detection kit (Kanglang Biological Technology Co., Ltd. Shanghai, CHN). The blood samples were incubated and centrifuged to obtain sera. The sera were diluted 5× , and 50 μl of diluted serum was added to an enzyme coated plate, then incubated at 37 °C for 30 min. Subsequently, 50 μl conjugate reagent was added after washing, incubated again and washed. Then 50 μl chromogenic agent A and 50 μl chromogenic agent B were added, and the color was developed at 37 °C for 10 min in the dark. Finally, 50 μl termination solution was added to each well to terminate the reaction. The absorbance of each well was measured at 450 nm with a microplate analyzer (Bio-Rad, USA). Serum 25(OH)D was determined according to the instructions of the Cobas E 601 Electrochemiluminescence Immunoanalyzer (Roche, Inc., USA). The sera to be tested were diluted 5× , and 50 μl diluted serum was incubated successively with pretreatment reagent, ruthenium-labeled vitamin D binding protein, streptavidin coated magnetic beads and biotin labeled 25(OH)D. After incubation, the reaction liquid was transferred into the measuring chamber, and the luminous intensity was measured with a photomultiplier. The results were obtained on the basis of the calibration curve of the detector.

### Isolation and culture of primary HSCs in BA

Fresh liver tissue samples (50 mg each) from patients with BA were washed with HBSS solution three times and cut into pieces. Pronase/collagenase digestion solution containing 1% DNase was added, and the digestion proceeded for 30 min at 37 °C. The digested cell suspensions were filtered through a 70 μm filter membrane, centrifuged and washed twice, then resuspended in 10 ml GBSS/B solution containing 1% DNase. Subsequently, 5 ml of Nycodenz solution was added. After mixing, 1 ml of Nycodenz solution was slowly added into the Cell-Nycodenz suspension, and centrifuged at 1380 g at 4 °C for 17 min. After centrifugation, a layer of white cells was visible between the Cell-Nycodenz and GBSS/B. The intermediate layer was absorbed and resuspended with GBSS/B solution. The BA primary HSCs were obtained by further centrifugation. The primary HSCs were resuspended in DMEM containing 20% FBS and penicillin (100 U/ml)-streptomycin (0.1 mg/ml) solution and incubated at 37 °C under 5% CO_2_ for 24 h. The cells were subsequently washed with PBS three times, and the culture solution was changed every 2 days. Passaging was performed when the cell density reached 90%.

### Establishment of a co-culture system

Hepatocytes QSG-7701 were evenly plated in the upper chamber of a six-well Transwell plate (CoStar Inc., USA) at a concentration of 4 × 10^5^/well, and culture medium containing the corresponding lentivirus (Nuobai Biological Technology Co., Ltd, Shanghai, CHN) was added for each transfection group. After transfection for 12 h, the culture medium containing virus was discarded. RPML 1640 culture medium containing 10% fetal bovine serum was replaced for further culturing. Subsequently, the culture medium was replaced every 24 h, and serum-free medium was used to replace the medium 72 h later for 12 h starvation treatment. The primary HSCs were plated in the lower chamber of the Transwell plate at a density of 30%. The culture medium in the upper chamber was supplemented with 1 μM VD and 10 ng/ ml TGF-β1 to induce fibrosis of HSCs. QSG-7701 hepatocytes were indirectly co-cultured with BA primary HSCs (separated by a 0.4 μm semi-permeable membrane) for 24 h. The primary HSCs in the lower chamber were finally collected for detection and analysis of the liver fibrosis related factors.

### Quantitative real-time polymerase chain reaction (qPCR)

The relative expression of CYP2R1, CYP27A1 and VDR in human liver samples; TGF-β1, Col-1α1 and α-SMA in primary HSCs under different culture conditions; and TGF-β1, Col-1α1, TIMP-1 and MMP-2 in liver tissue from mice infected with AAVs was assessed by qPCR. mRNAs were extracted from 30 mg liver tissue or 1 × 10^6^ primary HSCs with a Total RNA Extraction Kit (Solarbio Science & Technology Co., Ltd, Beijing, CHN) according to the manufacturer’s recommendations. The extracted genetic material was subjected to reverse transcription with random primers by using a PrimeScript RT reagent Kit (Takara, Biotechnology Co., Ltd.). cDNA was amplified by PCR with a KAPA 2G Robust HotStart ReadyMix PCR Kit (Kapa Biosystems, Roche, Switzerland). The PCR primers were designed in Primer Premier 7.0 and synthesized by Sangon Bioengineering (Shanghai) Co., Ltd. PCR was performed under the following conditions: initial denaturation at 95 °C for 10 min, followed by 40 cycles of 95 °C for 5 s, 60 °C for 30 s, 72 °C for 35 s, and 60 °C for 10 min for a final extension. The 2^−ΔΔC(T)^ method was used to analyze the relative changes in gene expression.

### Western blot assays

The differences in mRNA levels detected by qPCR were verified at the protein level by western blot assays. Liver tissue homogenates or HSCs were collected and lysed on ice for 30 min. After ultrasonication, the samples were centrifuged at 12000 g for 30 min, and the supernatant was collected. Total protein concentrations were determined with a BCA Protein Concentration Assay Kit (Enhanced) P0010S (Biyuntian Biotechnology Institute, Nantong, CHN). Equal amounts of samples were separated on 12% SDS–polyacrylamide gels at 80 V for 30 min and 110 V for 60 min. The protein bands were transferred to nitrocellulose membranes at 350 mA for 60 min, and the membranes were blocked at room temperature for 120 min. The nitrocellulose membranes were incubated with primary antibodies specific for CYP27A1 (1:1000), CYP2R1 (1:1000), TGF β1 (1:2000), collagen1 (1:1000), TIMP1 (1:1000) and MMP2 (1:1000) (Abcam Company, UK) at 4 °C overnight. Then the nitrocellulose membranes were washed with Tris-buffered saline and Tween 20 solution. The nitrocellulose membranes were incubated with IgG-HRP secondary antibody (1:5000) at room temperature for 60 min. Enhanced chemiluminescence assays were then performed. The gray levels of the protein bands were analyzed with Image-Pro Plus (version 7.0, Media Cybernetics, US).

### Immunohistochemical assays

Liver tissues were obtained from patients with BA and immunohistochemical staining was performed on formalin-fixed tissue sections. Serial paraffin-embedded sections were placed in citrate buffer (pH6.0) and boiled in a microwave oven for 15 min. Sections were treated with 3% hydrogen peroxide for10 min at room temperature and blocked with 5% BSA for 30 min. Sections were stained with rabbit anti-CYP27A1 and rabbit anti-CYP2R1 antibodies (1:200, Abcam, UK) overnight, followed by incubation biotin labeled secondary antibodies for 30 min and diaminobenzidine (DAB) for 1 min. Immunostaining results were evaluated by calculation of the mean density with Image-Pro Plus (version 7.0, Media Cybernetics, US).

### CCK-8 experiments

A total of 6000 HSCs were seeded in 96-well plates containing medium with or without 200 nM calcitriol. Then 10 μl CCK8 reagent was added at 0 h, 24 h, 48 h or 72 h. After the plates were incubated for 2 more hours, the absorbance at 450 nm was measured with a microplate analyzer (Biochrom Anthos, UK). The cell proliferation inhibition rate was calculated as follows: IOD at each time point/IOD at 0 h–1.

### Transwell cell migration assays

The upper chamber of a Transwell system was seeded with 200 μl HSC suspension at a density of 1 × 10^5^/ml in serum-free medium, and the lower chamber was filled with 600 μl complete medium with or without 200 nM calcitriol. After 24 h of culture, the lower cells were fixed with 4% paraformaldehyde for 30 min and stained with 1% crystal violet for 10 min. The plates were observed under a microscope and counted in five fields.

### Statistical analysis

The statistical software SPSS (version 20.0) was used for data analysis, and SPSS software and GraphPad Prism 8 software were used to produce charts. The measurement data were first tested for normality, and data with a normal distribution were expressed as the mean ± standard error of the mean (SEM). The t-test was used for comparison between two groups, one-way ANOVA was used for comparison between multiple groups of data, and Bonferroni correction was used for pairwise comparison. The statistical significance was set at *P* < 0.05.

All clinical studies and animal experimental protocols in this study were approved by the Ethics Committee of Children’s Hospital of Fudan University. All methods used in this study were performed in accordance with the relevant guidelines and regulations both for animal experiment and human involvement. Informed consent was obtained from the parents or legal guardians of all involved participants.

## Results

### Basic clinical data

Ten children were enrolled in the BA and control groups. The general conditions and the results of laboratory examination of the children are listed in Table 1. There was no significant difference in age, sex, body weight, hemoglobin, international standardized ratio, alkaline phosphatase, and glutamyl transpeptidase between the two groups. The total bilirubin, direct bilirubin, alanine transaminase, and aspartate transaminase in the BA group were higher than in the control group (*P* < 0.05). Creatinine was slightly lower in the BA group than in the control group (*P* < 0.05).

**Table 1:** General information and laboratory test results of children enrolled in the clinical study.

	BA group (n = 10)	Control group (n = 10)	*P* value
Age (days)	59.4 ± 17.4	57.6 ± 32.0	0.878
Gender (M/F)	3/7	5/5	0.650
Weight (kg)	4.6 ± 0.3	4.7 ± 0.9	0.846
TB (μmol/L)	175.5 ± 66.9	87.3 ± 54.1	0.005*
DB (μmol/L)	115.3 ± 47.4	41.2 ± 24.1	0.001*
ALT (IU/L)	186.0 ± 186.9	60.9 ± 41.9	0.044*
AST (IU/L)	242.7 ± 166.5	81.1 ± 45.3	0.014*
ALP (IU/L)	587.4 ± 289.7	533.4 ± 242.4	0.600
GGT (IU/L)	454.3 ± 289.7	544.9 ± 440.2	0.594
Hb (g/L)	99.2 ± 11.8	105.8 ± 18.0	0.350
INR	1.0 ± 0.1	1.1 ± 0.2	0.521
Crea (μmol/L)	19.4 ± 1.7	24.9 ± 5.5	0.012*

*TB* total bilirubin, *DB* direct bilirubin, *ALT* alanine aminotransferasem *AST* aspartate transaminase, *ALP* alkaline phosphatase, *GGT* γ-glutamyl transpeptidase, *Hb* hemoglobin, *INR* international standardized ratio, *CREA* creatinine.

Asterisks denote statistically significant differences (Student’s t test: *p < 0.05).

### Level of 25(OH)D and ratio of 25(OH)D/vitamin D in the BA group were significantly lower than in the control group

**Figure 1:**
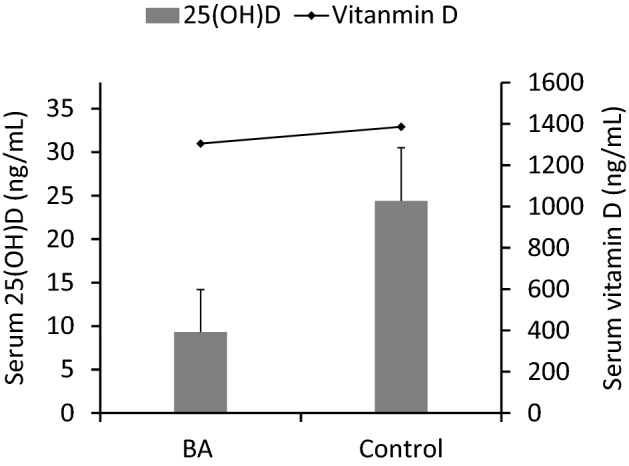
Serum levels of vitamin D in the BA and control groups were similar, while 25(OH)D level in the BA group was significantly lower than in the control group, thus, suggesting that conversion of vitamin D to 25(OH)D was impaired in children with BA.

The serum levels of vitamin D were detected by ELISA: 1386.0 ± 153.1 and 1305.3 ± 244.8 ng/mL in the control and BA groups, respectively. 25(OH)D was detected by electrochemiluminescence. The level of 25(OH)D in the control and BA groups was 24.3 ± 6.1 and 9.3 ± 4.9 ng/mL, respectively. 25(OH)D level in the BA group was significantly lower than that in the control group (*P* < 0.001) (Fig. 1). The ratio of 25(OH)D to vitamin D in serum was used to evaluate the capacity of 25 hydroxylation during vitamin D activation. The ratio of 25(OH)D/vitamin D was 1.75 ± 0.44% in the control group, which was significantly higher than 0.72 ± 0.40% in the BA group (*P* < 0.001). This suggested that the 25 hydroxylation ability of vitamin D in the BA group was impaired.

### Expression of CYP2R1 and CYP27A1 in liver tissue of children with BA group was significantly lower than in the control group

**Figure 2:**
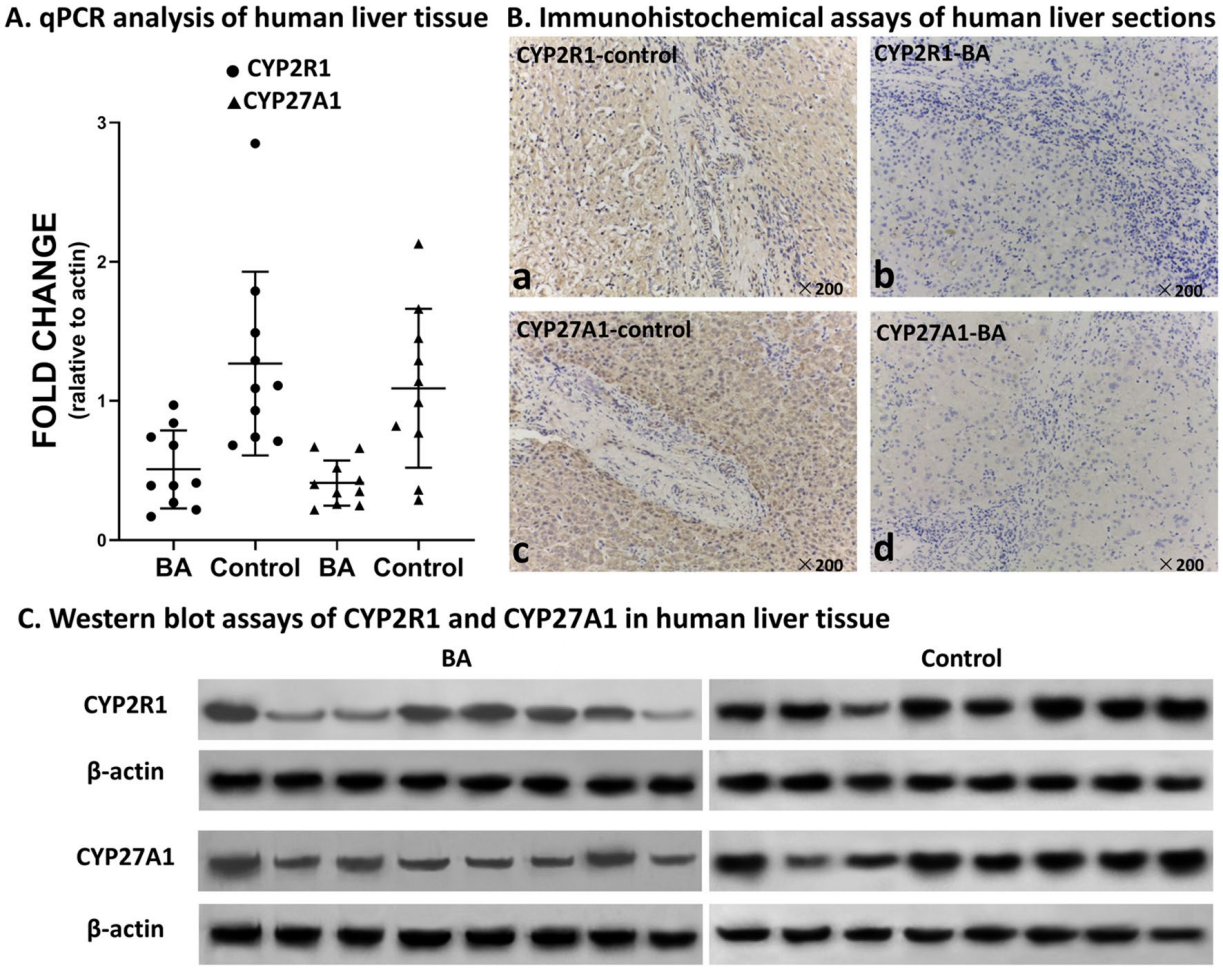
Expression of 25-hydroxylase CYP2R1 and CYP27A1 in liver tissue of BA and control groups. (**A**) Dot plot of mRNA levels of CYP2R1 and CYP27A1. (**B**) The expression of CYP2R1 and CYP27A1 in the BA and control groups detected by immunohistochemistry. Brown staining represents expression of CYP2R1 and CYP27A1. a and b show expression of CYP2R1 in the control and BA groups, and c and d show expression of CYP2R1 in the control and BA groups, respectively. (**C**) The expression of CYP2R1 and CYP27A1 in the BA and control groups detected by western blotting.

To verify the vitamin D activation disorder in BA, we detected the expression of CYP2R1 and CYP27A1, which are important hydroxylases that convert vitamin D to 25(OH)D in the liver. qPCR showed that mRNA expression of CYP2R1 and CYP27A1 in the BA group was significantly lower than in the control group (CYP2R1: 0.51 ± 0.28 vs. 1.27 ± 0.63, *P* = 0.004; CYP27A1: 0.41 ± 0.16 vs. 1.09 ± 0.57, *P* = 0.004) (Fig. 2), and no significant difference was detected in the expression of VDR mRNA between the two groups (0.61 ± 0.39 vs. 0.53 ± 0.43, *P* = 0.698). Immunohistochemical analysis of liver tissue sections showed that the expression of CYP2R1 and CYP27A1 proteins were strongly positive in liver tissue of the control group, but slightly positive in the BA group. The difference was significant: integrated option density (IOD)/area CYP2R1: 0.036 ± 0.020 vs. 0.069 ± 0.011, *P* = 0.002; CYP27A1: 0.033 ± 0.016 vs. 0.071 ± 0.015, *P* < 0.001) (Fig. 2). Western blotting verified the expression of CYP2R1 and CYP27A1, which confirmed that expression of CYP2R1 and CYP27A1 proteins in liver tissues of patients with BA was significantly lower than in the control group (CYP2R1: 0.67 ± 0.28 vs. 1.13 ± 0.43, *P* = 0.026; CYP27A1: 0.41 ± 0.15 vs. 0.68 ± 0.27, *P* = 0.035) (Fig. 2).

### Culture and identification of primary HSCs of patients with BA

**Figure 3:**
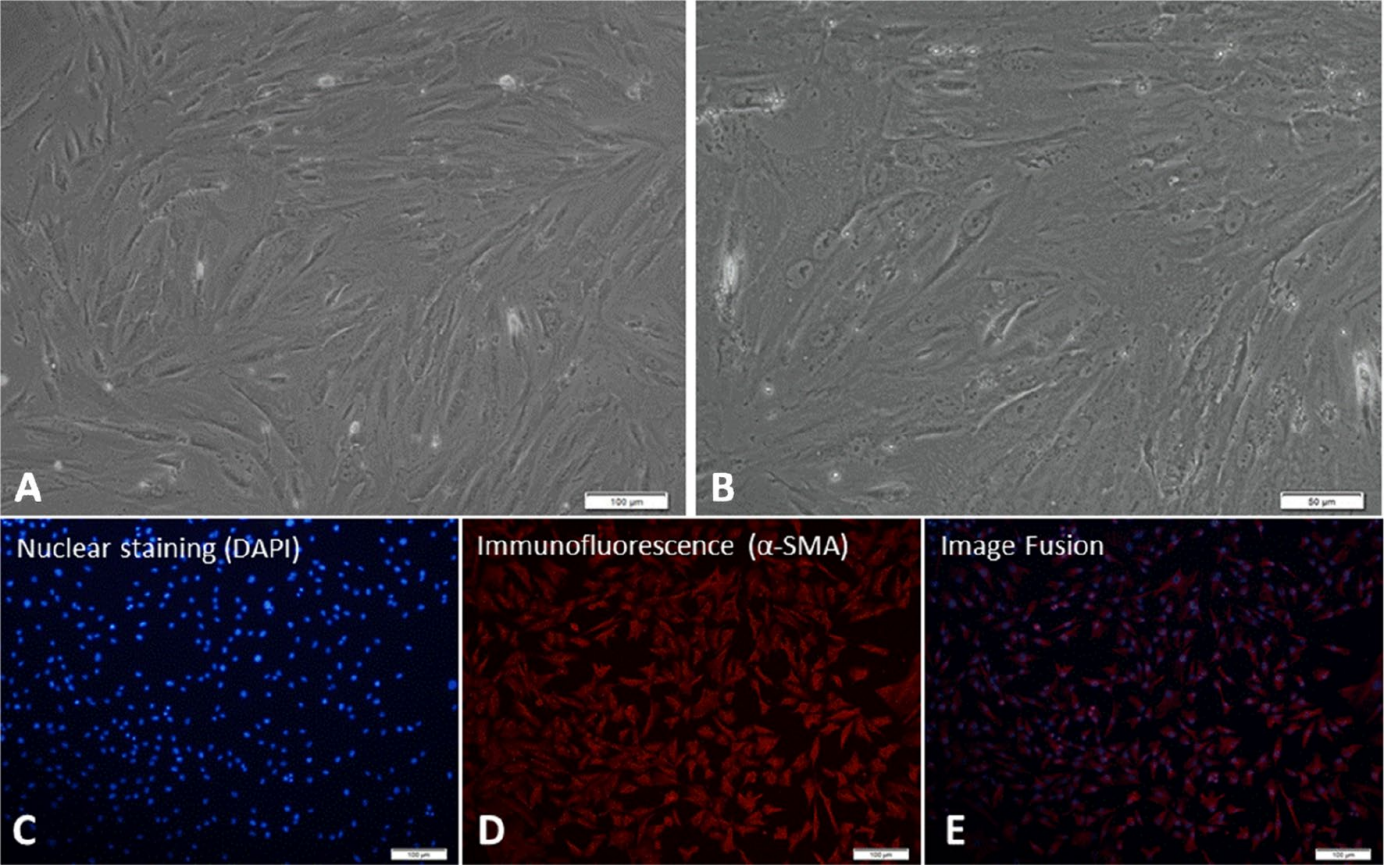
(**A**) Primary HSCs isolated from BA patients showed typical morphology (100×) after 3 days of culture. (**B**) Local magnification shows intracellular lipid droplets (200×). Expression of α-SMA in primary HSCs of BA was detected by immunofluorescence assay. (**C**) Nuclear staining (DAPI). (**D**) Cytoplasm showed red fluorescence indicating positive α-SMA expression. (**E**) Fusion images: the positive rate of α-SMA was 97% after counting.

Primary HSCs isolated by density gradient centrifugation were spherical cells with high refraction under inverted microscopy, and lipid droplets were clearly seen in the cytoplasm. After 24 h of culture, most of the cells began to grow adherently, and a few cells began to extend their tentacles. After 2–3 days of culture, most of the cells showed polycephalous pseudopods or typical HSC morphology (Fig. 3). On the seventh day of culture, the lipid droplets in the cytoplasm disappeared completely, and the cells became spindle-shaped. After 12–14 days of culture, the cells grew into monolayers. Immunofluorescence assays showed that the percentage of α-SMA-positive cells was 97% on day 7 of culture (Fig. 3).

### Calcitriol inhibits expression of fibrosis-related genes, proliferation and migration of primary HSCs from patients with BA in vitro

Primary HSCs of patients with BA were cultured in vitro with calcitriol at 100, 200 and 400 nM for 24 h. qPCR indicated that expression of TGF-β1, Col-1α1 and α-SMA was decreased after addition of calcitriol, and the decrease was most significant with 200 nM calcitriol (Table 2).

**Table 2:** Effects of calcitriol at different concentrations on the mRNA expression of TGF-β1, Col-1α1 and α-SMA in pHSC.

	Untreated group	100 nM group	200 nM group	400 nM group
TGF-β1	1.00 ± 0.27	0.73 ± 0. 16*	0.53 ± 0. 15*	0.56 ± 0. 18*
Col-1α1	1.00 ± 0.16	0.90 ± 0.22*	0.71 ± 0. 14*	0.76 ± 0.11*
α-SMA	1.00 ± 0.17	0.97 ± 0.28	0.59 ± 0.06*	0.66 ± 0.20*

*Compared with the untreated group, *P* < 0.05.

**Figure 4:**
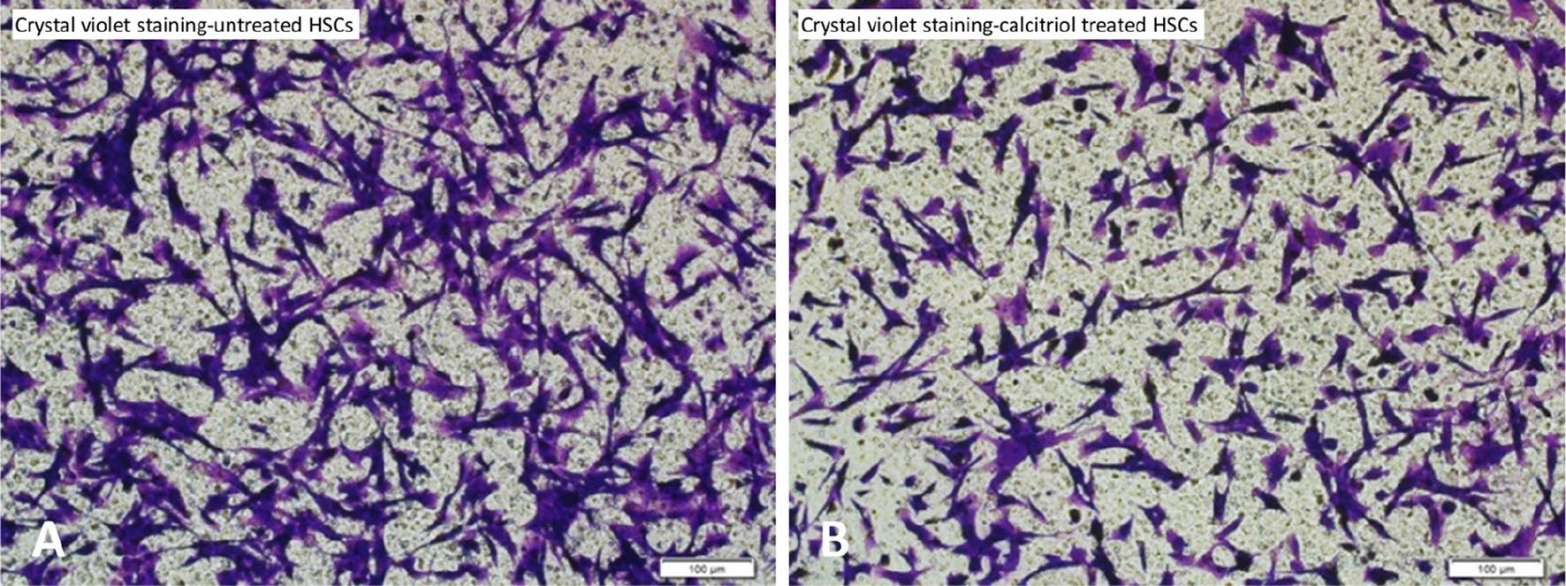
The Transwell assay showed that the migrated cell count of the untreated group (**A**) was higher than that of the 200 nM calcitriol treatment group (**B**) (*P* < 0.01).

The proliferation of primary HSCs treated with 200 nM calcitriol for 24, 48 and 72 h was lower than that of untreated HSCs (0.34 ± 0.13 vs. 0.46 ± 0.09, *P* < 0.01; 0.88 ± 0.15 vs. 1.01 ± 0.11, *P* < 0.01; 1.51 ± 0.13 vs. 1.73 ± 0.10, *P* < 0.01). The Transwell assay showed that the migrated cell count of the untreated group was 382 ± 51, and that of the 200 nM calcitriol group was 223 ± 76, which was a significant difference (*P* < 0.01) (Fig. 4). These results suggest that calcitriol can inhibit the proliferation and migration of HSCs.

### Construction of CYP2R1 lentivirus and validation of hepatocyte transfection effect

CYP2R1-overexpression lentivirus (CL1674-PDS237_PL-CMV-GFP-CYP2R1) and CYP2R1-interference lentivirus (CL1676-PDS126_PL6.3-shRNA-GFP-CYP2R1-932) vectors were constructed with titers of 4.5 × 10^8^ TU/mL. QSG-7701 hepatocytes were transfected with CYP2R1-overexpression lentivirus, CYP2R1-interference lentivirus or negative control lentivirus. qPCR showed that the expression of CYP2R1 mRNA in the CYP2R1 overexpression group was significantly higher than in the negative control group (9.39 ± 0.34 vs. 1.0 ± 0.04, *P* < 0.001), and expression in the CYP2R1-interference group was significantly lower than in the negative control group (0.37 ± 0.06 vs. 1.0 ± 0.04, *P* < 0.001). These results indicated that CYP2R1-overexpression lentivirus increased expression of CYP2R1, while CYP2R1-interference lentivirus reduced expression of CYP2R1.

### Upregulation of expression of CYP2R1 in hepatocytes reduced mRNA and protein expression of fibrosis-related factors in cocultured primary HSCs of BA

**Figure 5:**
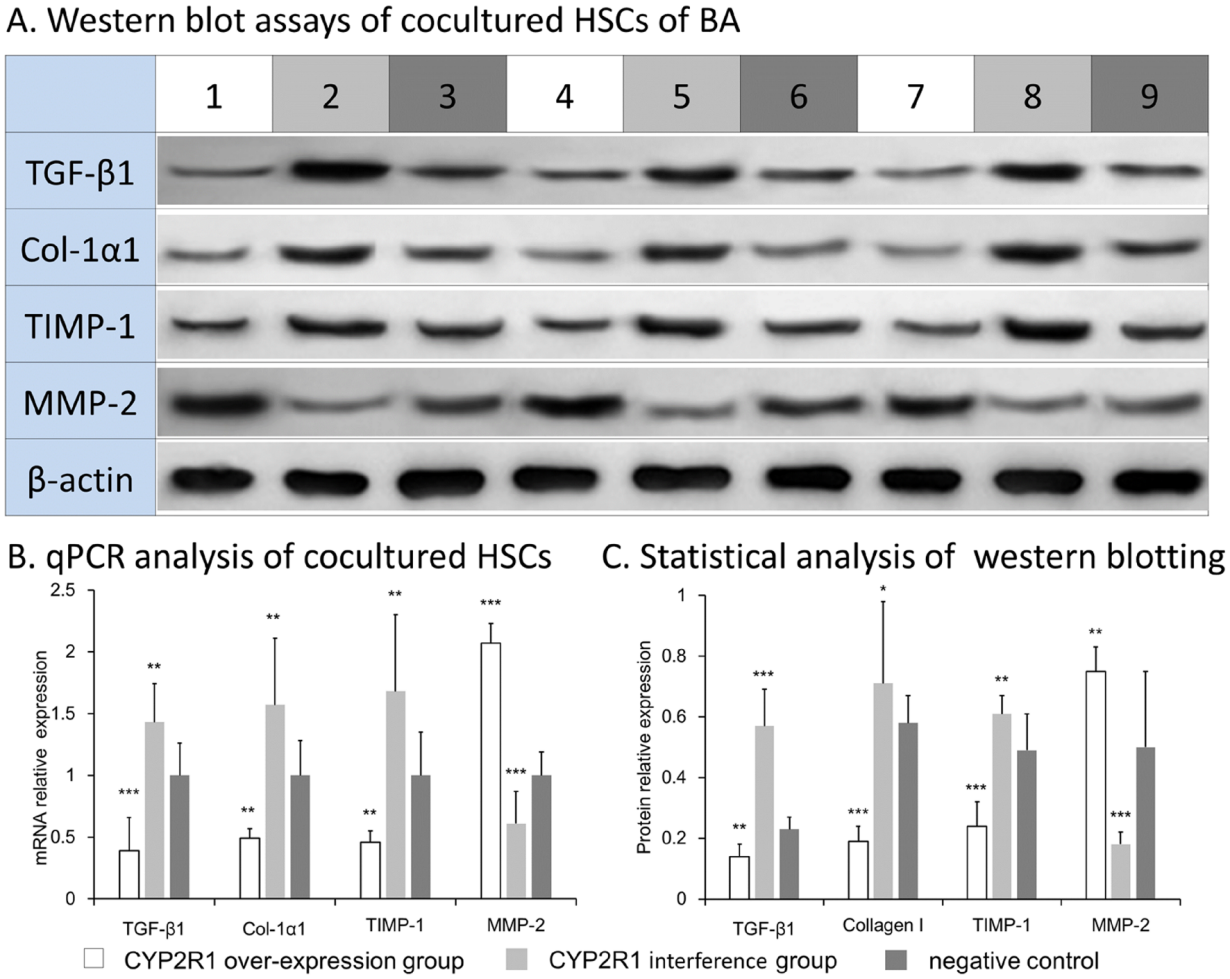
Regulation of expression of CYP2R1 in hepatocytes can change mRNA and protein expression of fibrosis-related factors in cocultured primary HSCs of BA. (**A**) protein expression of fibrosis-related factors in CYP2R1-overexpression group (columns 1, 4 and 7), CYP2R1-interference group (columns 2, 5 and 8) and negative control group (columns 3, 6 and 9). (**B,C**) statistical results of qPCR and western blotting detection of fibrosis-related factors in each intervention group.

Primary HSCs of patients with BA were cocultured with CYP2R1-overexpression QSG-7701 hepatocytes, CYP2R1-interference QSG-7701 hepatocytes, or QSG-7701 hepatocytes (negative control). After incubation for 48 h, primary HSCs of BA in the coculture system were collected and assayed by qPCR. mRNA expression of TGF-β1, Col-1α1 and TIMP-1 in the CYP2R1 overexpression group was lower than in the negative control group (*P* < 0.001), and higher in the CYP2R1 interference group than in the negative control group (*P* < 0.01). mRNA expression of MMP-2 in the CYP2R1-overexpression group was higher than in the negative control group (*P* < 0.001), and was lower in the CYP2R1-interference group than in the negative control group (*P* < 0.01) (Fig. 5). Western blotting showed that expression of these liver-fibrosis-related factors at the protein level also complied with this trend (Fig. 5). These results indicated that overexpression of CYP2R1 in hepatocytes decreased expression of TGF-β1, Col-1α1 and TIMP-1, and increased expression of MMP-2 in HSCs, while low expression of CYP2R1 in hepatocytes increased expression of TGF-β1, Col-1α1 and TIMP-1, and decreased expression of MMP-2.

### Downregulation of CYP2R1 expression in liver of mice with biliary obstruction aggravated cirrhosis

**Figure 6:**
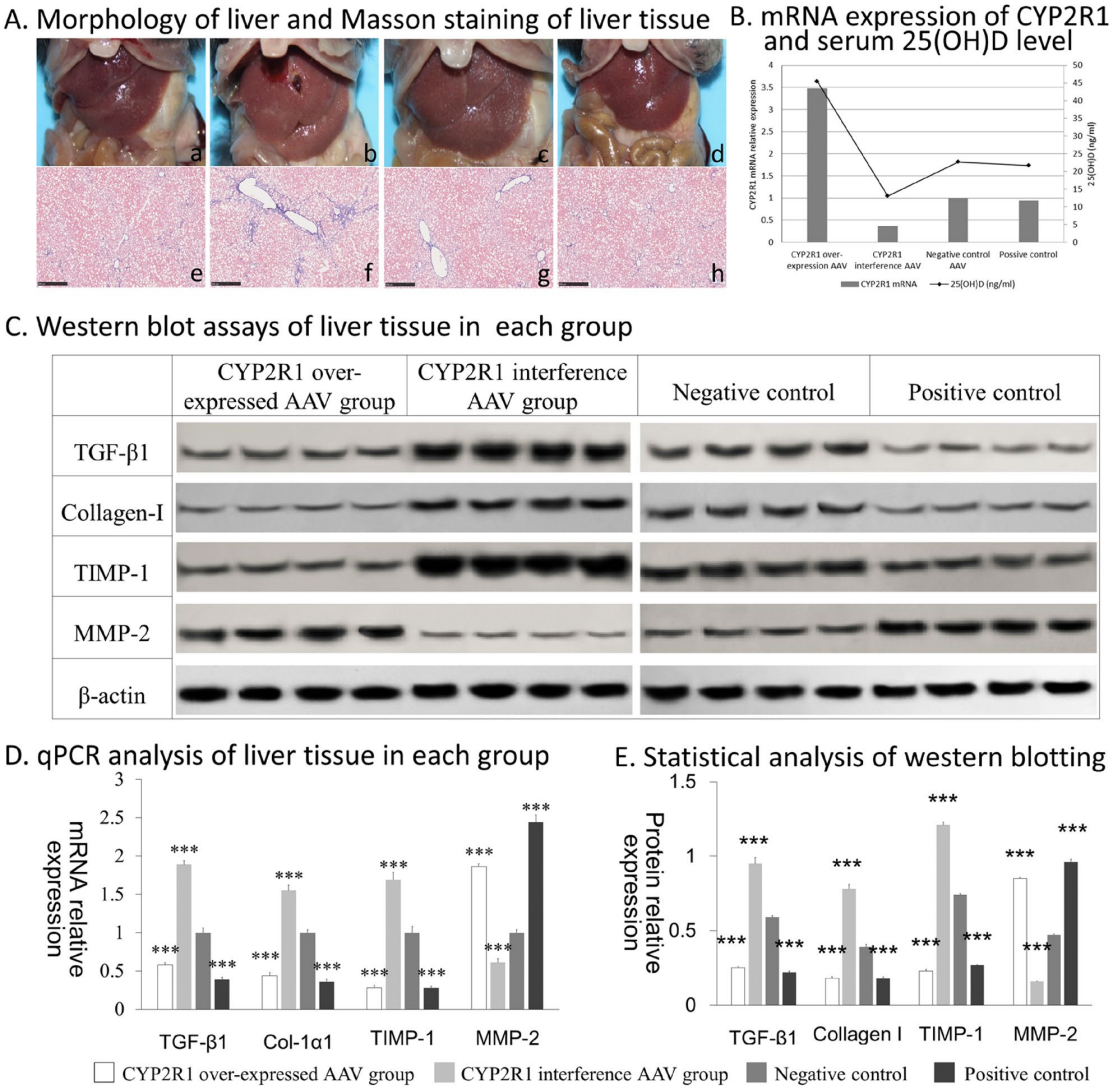
(**A**) morphology of liver and Masson staining of liver tissue: the liver of the CYP2R1-overexpression AAV group (a) and positive control group (d) was ruddy, soft, smooth and the liver capsule was not tense. In the CYP2R1-interference AAV group (b), the liver was enlarged with dark color, hard texture and nodular protuberance on the surface. The liver of negative control group (c) was enlarged, with tight, brown and slightly hard liver capsule. Masson staining of liver tissue of mice showed that there were more blue collagen fibers deposited in liver tissue of the CYP2R1-interference group (f), extending outward from around the vascular region, and the fibers were thicker and the staining was darker. The CYP2R1-overexpression AAV group (a) and positive control group (g) showed less blue collagen deposition in liver tissues. (**B**) mRNA expression of CYP2R1 and serum 25(OH)D level in each intervention group. (**C**) protein level of the liver-fibrosis-related factors in each intervention group detected by western blotting. (**D**,**E**) statistical results of qPCR and western blotting detection of liver-fibrosis-related factors in each intervention group.

CYP2R1-overexpression AAV (CL1831-PDS272_PAAV-CMV-GFP-CYP2R1) and CYP2R1-interference AAV (CL1842-PAAV-U6-CYP2R1-shRNA-932) vectors were constructed with titers of 1 × 10^12^ VP/ mL. The first day after bile duct ligation of C57BL/6 mice, CYP2R1-overexpression AAV, CYP2R1-interference AAV, negative control AAV or 2 μg/kg calcitriol (positive control) was injected. The mice were fed for 2 more weeks. The liver morphology was observed as follows: the liver of the negative control group was enlarged, with tight, brown and a slightly hard liver capsule; the liver of the CYP2R1-overexpression AAV group and the positive control group was ruddy, soft, smooth and the liver capsule was not tight. In the CYP2R1-interference AAV group, the liver was enlarged with dark color, hard texture and nodular protuberance on the surface. Masson staining of liver tissue showed that there were more blue collagen fibers deposited in liver tissue of the CYP2R1-interference group, extending outward from portal areas and their surroundings, and the fibers were thicker and the staining was darker than in the other groups. The CYP2R1-overexpression group and calcitriol-positive control group showed less blue collagen deposition in liver tissue (Fig. 6).

### Overexpression or interference of the CYP2R1 gene affected 25(OH)D level through changing CYP2R1 expression

qPCR showed that the expression of CYP2R1 mRNA in the CYP2R1-overexpression AAV group was higher than that in the negative control group (*P* < 0.001), and expression in the CYP2R1-interference AAV group was lower than in the negative control group (*P* < 0.001). This indicated that gene transfection successfully altered expression of CYP2R1. The 25(OH)D level of mice in the CYP2R1-overexpression AAV group was higher than in the negative control group (*P* < 0.001), and the 25(OH)D level in the CYP2R1-interference AAV group was lower than in the negative control group (*P* < 0.01). This shows that CYP2R1 overexpression can lead to an increase in 25(OH)D levels, and downregulation of CYP2R1 can lead to a decrease in 25(OH)D levels (Fig. 6).

### Overexpression or interference of the CYP2R1 gene affected expression of mRNA and protein levels of liver fibrosis-related factors in mice with biliary ligation

qPCR showed that mRNA expression of TGF-β1, Col-1α1 and TIMP-1 in the CYP2R1-overexpression AAV group and calcitriol-positive control group was lower than in the negative control group (*P* < 0.001), and expression in the CYP2R1-interference AAV group was higher than in the negative control group (*P* < 0.01). These results suggested that increasing expression of CYP2R1 or adding exogenous calcitriol reduced the expression of liver-fibrosis-related genes, whereas decreasing expression of CYP2R1 increased expression of liver-fibrosis-related genes. In contrast, the expression of antifibrosis factor MMP-2 mRNA was higher in the CYP2R1-overexpression AAV group and calcitriol positive control group than in the negative control group (*P* < 0.001). Expression in the CYP2R1-interference group was lower than in the negative control group (*P* < 0.001), indicating that CYP2R1 or exogenous calcitriol increased the expression of MMP-2 (Fig. 6). Western blotting verified the expression at the protein level of the above hepatic-fibrosis-related factors, and showed that the changes in each protein were consistent with the mRNA levels (Fig. 6).

## Discussion

BA is the most common biliary obstructive disease in infants, and the most common cause of jaundice, with an incidence of 1/8000–18,000^1,2^. BA is a fibrous inflammatory disease of the intrahepatic and extrahepatic bile duct tree, characterized by intrahepatic and extrahepatic bile duct atresia and progressive hepatic fibrosis. The liver fibrosis of BA is earlier and more severe than any other infant cholestatic disease^3–7^; therefore, the presence of cirrhosis is often used for differential diagnosis of BA. Liver fibrosis may be the primary pathogenic factor in the pathological mechanism of BA, rather than biliary cirrhosis secondary to cholestasis alone. Although liver fibrosis has traditionally been considered an irreversible process, it is now believed that liver fibrosis is a reversible pathological event, especially in children, if there is an underlying mechanism for timely intervention to prevent liver fibrosis. Therefore, the treatment of liver fibrosis after HPE is important and deserves further study to avoid liver transplantation. It is now gradually realized that without a clear understanding of the mechanism and influencing factors of liver fibrosis, it is difficult to achieve further breakthroughs in the treatment of autologous liver of BA. The mechanism of liver fibrosis in BA has been studied extensively, mainly focusing on immune regulation disorder^8–10^, viral infection^11,12^, and excessive release of inflammatory factors^1^. These factors may play a role in aggravating cirrhosis to some extent or in some patients, but there is still no consensus on the mechanism of the formation or aggravation of cirrhosis in BA, which may be a result of the combined action of multiple factors.

The main source of vitamin D is the daily diet, and vitamin D absorbed from food is first converted to 25(OH)D by hydroxylation of CYP2R1 and CYP27A1 in the liver. It is then hydroxylated to 1,25,(OH)_2_D in the presence of CYP27B1 in the kidneys. Both 25(OH)D and 1,25,(OH)_2_D have biological activity. 1,25,(OH)_2_D is the final active product of vitamin D metabolism, which has higher biological activity than 25(OH)D has. Activated vitamin D can bind to the vitamin D receptor (VDR) and then play a biological role. VDR is a member of the nuclear receptor superfamily, and its classical biological effects are mainly to maintain calcium homeostasis and calcium deposition in bone. In recent years, vitamin D has been found to have antifibrotic effects in lung, kidney and other tissues^13–18^. VDR is not expressed in liver tissue, so the role of vitamin D in liver fibrosis has not been studied in depth for a long time. However, the latest studies have found that VDR is expressed in nonparenchymal liver cells^19^, such as HSCs, which suggests that vitamin D is a regulatory factor in liver fibrosis.

Vitamin D deficiency is common in liver diseases, especially in the case of cirrhosis, but it is difficult to determine the causal relationship between vitamin D deficiency and liver diseases. It is generally believed that absorption and activation of vitamin D are impaired due to liver dysfunction in cirrhosis, although there are few studies on the effect of vitamin D deficiency on cirrhosis. The studies of patients with chronic liver disease have shown that low serum 25(OH)D is strongly associated with severe cirrhosis and low responsiveness to treatment^20–23^. Vitamin D deficiency can aggravate the degree of liver fibrosis in patients with liver disease^20,23^, while supplementation with vitamin D can improve liver fibrosis in patients with chronic liver disease^24,25^. Vitamin D deficiency or VDR gene polymorphism increases the risk of cirrhosis^26,27^. These results suggest that vitamin D plays an important role in the occurrence of liver fibrosis. In vitro and in vivo experimental studies have found that vitamin D can reduce the proliferation of HSCs, inhibit expression of profibrosis genes such as Col-1α1 and TIMP-1, and promote expression of antifibrosis genes such as MMP9^28–30^. VDR gene knockout can lead to primary liver fibrosis^31^. Based on the above studies, in the case of severe liver disease, there may be a vicious cycle of aggravation of liver fibrosis and impairment of vitamin D absorption and activation, which play a role in the progression of cirrhosis^32^.

As early as 1979, the lack of 25(OH)D in children with BA was noted by Japanese researchers^33^. However, in the following decades, this issue did not attract widespread attention or in-depth research, and clinicians took it for granted that it was due to the absorption deficiency of fat-soluble vitamins caused by reduced bile. In recent years, as more studies have gradually revealed the internal relationship between vitamin D deficiency and liver fibrosis, researchers have begun to pay attention to this problem. Ng et al.^34^ detected the pre-HPE 25(OH)D level in 92 children with BA, and found that 98.9% of the children had 25(OH)D deficiency or insufficiency, and only one (1.1%) had a normal level of 25(OH)D. Zhuang et al.^35^ examined the 25(OH)D level before HPE of 161 BA cases, and found that 96.3% of the children had 25(OH)D deficiency or insufficiency. However, these studies confirmed deficiency of 25(OH)D in children with BA, but did not further investigate the cause of that deficiency and its effect on liver lesions. Considering that the current clinical evaluation of vitamin D status is mostly through the detection of serum 25(OH)D level, and the current vitamin D supplement program is mostly oral vitamin D, oral intake of vitamin D cannot improve 25(OH)D deficiency, and serum 25(OH)D level does not reflect the extent of vitamin D deficiency in cases of vitamin D hydroxylation disorders.

In order to comprehensively evaluate the levels of vitamin D and 25(OH)D in vivo, we detected the serum levels of vitamin D and 25(OH)D in the BA and control groups. The level of vitamin D in BA was lower than in the control group, while the level of 25(OH)D was significantly lower than in the control group. The ratio of 25(OH)D/vitamin D was used to reflect the efficiency of vitamin D transformation to 25(OH)D, and the results also showed that there was a significant reduction in vitamin D activation rate in the BA group. It has been shown that CYP2R1 and CYP27A1 are key enzymes in the first step of hydroxylation of vitamin D, in which CYP2R1 plays a major role in the transformation of vitamin D to 25(OH)D^36–38^. CYP2R1 is expressed only in the liver, so that is the only site for the initial hydroxylation of vitamin D. In the present study, CYP2R1 expression was detected in the liver of children with BA, and the results showed that CYP2R1 expression was significantly decreased. Therefore, the decreased expression of CYP2R1 was the main cause of 25(OH)D deficiency, rather than vitamin D malabsorption, which could explain why 25(OH)D deficiency was difficult to be corrected by oral supplementation of vitamin D in clinical practice.

As previously mentioned, active vitamin D deficiency can aggravate liver fibrosis in liver disease, and children with BA have significant 25(OH)D deficiency and vitamin D activation disorder. Whether the active vitamin D deficiency and activation disorder in BA are involved in the pathological process of liver fibrosis has not been researched to date, even though a correlation between 25(OH)D deficiency and liver fibrosis in BA has been confirmed in several clinical studies. Zhuang et al.^35^ examined the level of 25(OH)D in 161 children with BA and staged liver fibrosis in pathological specimens, and found that 25(OH)D level was negatively correlated with the stage of liver fibrosis. Peng et al.^39^ studied the correlation between 25(OH)D level and liver shear wave elastography in 33 children with BA after HPE, and found that 25(OH)VD level was negatively correlated with liver fibrosis severity.

Activation of HSCs and excessive deposition of extracellular matrix are two important pathological processes in liver fibrosis. Although the exact mechanism that regulates this process remains controversial, the TGF-β signaling pathway is considered to be one of the major pathways that promote the accumulation of fibrotic extracellular matrix^40^. Previous animal studies have confirmed that the binding of active vitamin D and VDR can regulate activity of the TGF-β signaling pathway in HSCs, thereby inhibiting expression of fibrosis genes and expression^28,31^ and deposition of types I and III collagen^30,41^. A recent study found that serum N-terminal peptide of procollagen III, an indicator of liver fibrosis, was negatively correlated with 25(OH)D in children with BA^35^. At present, there has been no study on the mechanism of 25(OH)D deficiency in BA, nor on the mechanism of the relationship with liver fibrosis. Studies on the mechanism of the effect of vitamin D on liver fibrosis have mostly used cell lines and animal models of chemical damage, which is different from the pathological process of BA. In the present study, the primary HSCs of BA were used for the first time to verify the role of vitamin D activation disorder in the process of liver fibrosis. Since early and severe liver fibrosis is an important pathological feature of BA, which is different from other cholestatic diseases, we adopted the animal model of biliary ligation to simulate the influence of vitamin D activation disorder on liver fibrosis in the case of cholestasis, which can reveal the role of vitamin D activation disorder in BA.

In our study, synthetic active vitamin D (calcitriol) was added to primary HSCs isolated from liver specimens from children with BA. The proliferation and migration of primary HSCs were decreased, along with expression of TGF-β, as well as Col-1α1, α-SMA and TIMP-1. Expression of CYP2R1 in hepatic cells was upregulated or downregulated by gene transfection and coculture with HSCs. The results showed that upregulation of CYP2R1 also inhibited expression of hepatic-fibrosis-related genes such as TGF-β1, Col-1α1 and TIMP-1, and promoted expression of antifibrosis genes such as MMP-2. The expression of CYP2R1 in mice with biliary tract ligation was altered by gene transfection, and increased expression of CYP2R1 in vivo could significantly inhibit expression of hepatic-fibrosis-related genes. Therefore, it can be concluded from this study that deficiency of active vitamin D in BA can promote the formation of liver fibrosis, and the main reason for this deficiency is deficiency of CYP2R1, which leads to disorder of vitamin D activation. This finding provides a new therapeutic target and theoretical basis for the antifibrosis treatment of BA. However, the molecular biological mechanism of CYP2R1 deficiency in BA remains unclear, and its regulatory mechanism needs to be further studied.

## Supplementary Information

Supplementary Information.
