# Supplementary material for: Effect and mechanism of vitamin D activation disorder on liver fibrosis in biliary atresia
Source: Sci Rep. 2021 Oct 6;11:19883. doi: 10.1038/s41598-021-99158-3 (PMC8494743; doi:10.1038/s41598-021-99158-3)
Supplement: Supplementary file 1 — Supplementary Information. [file 41598_2021_99158_MOESM1_ESM.docx]

**Table A.** Expression of fibrosis-related factors in primary HSCs of BA in the coculture system after regulating the CYP2R1 expression of hepatocytes

|  |  | CYP2R1 over-exression group | CYP2R1 interference group | Negative control group |
| --- | --- | --- | --- | --- |
| mRNA | TGF-β1 | 0.39±0.27 ^*^ | 1.43±0.31 ^*^ | 1.00±0.26 |
|  | Col-1α1 | 0.49±0.08 ^*^ | 1.57±0.54 ^*^ | 1.00±0.28 |
|  | TIMP-1 | 0.46±0.09 ^*^ | 1.68±0.62 ^*^ | 1.00±0.35 |
|  | MMP-2 | 2.07±0.16 ^*^ | 0.61±0.26 ^*^ | 1.00±0.19 |
| Protein | TGF-β1 | 0.14±0.04 ^*^ | 0.57±0.12 ^*^ | 0.23±0.04 |
|  | Collagen I | 0.19±0.05 ^*^ | 0.71±0.27 ^*^ | 0.58±0.09 |
|  | TIMP-1 | 0.24±0.08 ^*^ | 0.61±0.06 ^*^ | 0.49±0.12 |
|  | MMP-2 | 0.75±0.08^*^ | 0.18±0.04 ^*^ | 0.50±0.25 |

* Compared to the negative control group, P < 0.05.

**Table B.** Relative mRNA expression of CYP2R1 and 25(OH)D level in mice from different treatment groups

|  | CYP2R1 over-expression AAV group | CYP2R1 interference AAV group | Negative control AAV group | Calcitriol positive control group |
| --- | --- | --- | --- | --- |
| CYP2R1 mRNA | 3.48±0.63 ^*^ | 0.37±0.06 ^*^ | 1.00±0.22 | 0.94±0.18 |
| 25(OH)D (ng/ml) | 45.5±7.9 ^*^ | 13.1±0.65 ^*^ | 22.7±3.5 | 21.7±2.7 |

* Compared to the negative control AAV group, P < 0.05.

**Table C.** mRNA and protein expression of fibrosis-related factors in liver of mice with biliary ligation in different treatment groups

|  |  | CYP2R1 over-expression AAV group | CYP2R1 interference AAV group | Negative control AAV group | Calcitriol positive control group |
| --- | --- | --- | --- | --- | --- |
| mRNA | TGF-β1 | 0.58±0.12 ^*^ | 1.89±0.19 ^*^ | 1.00±0.23 | 0.39±0.10^*^ |
|  | col-1α1 | 0.44±0.15 ^*^ | 1.55±0.27 ^*^ | 1.00±0.15 | 0.36±0.11^*^ |
|  | TIMP-1 | 0.28±0.11 ^*^ | 1.69±0.39 ^*^ | 1.00±0.31 | 0.28±0.08^*^ |
|  | MMP-2 | 1.86±0.25 ^*^ | 0.61±0.18 ^*^ | 1.00±0.17 | 2.44±0.40^*^ |
| Protein | TGF-β1 | 0.25±0.04 ^*^ | 0.95±0.17 ^*^ | 0.59±0.05 | 0.22±0.04^*^ |
|  | Collagen-I | 0.18±0.03 ^*^ | 0.78±0.13 ^*^ | 0.39±0.08 | 0.18±0.03^*^ |
|  | TIMP-1 | 0.23±0.04 ^*^ | 1.21±0.09 ^*^ | 0.74±0.06 | 0.27±0.02^*^ |
|  | MMP-2 | 0.85±0.05 ^*^ | 0.16±0.02 ^*^ | 0.47±0.04 | 0.96±0.18^*^ |

* Compared to the negative control AAV group, P < 0.05.

**Supplemental images for Figure 2**

**
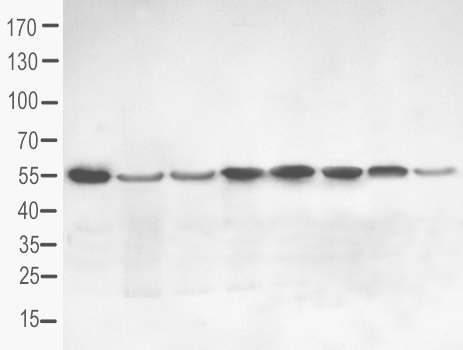
CYP2R1-BA**

**
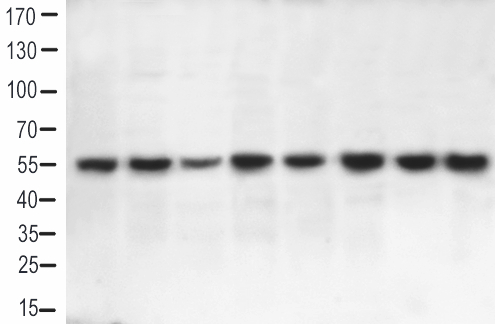
**

**CYP2R1-Control**

**CYP27A1-BA
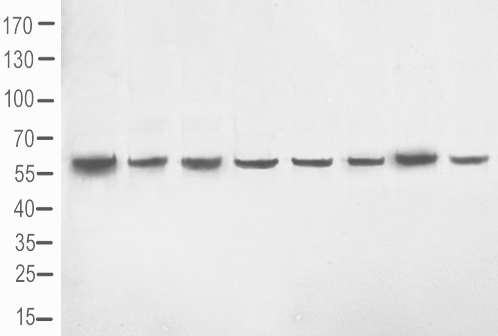
**


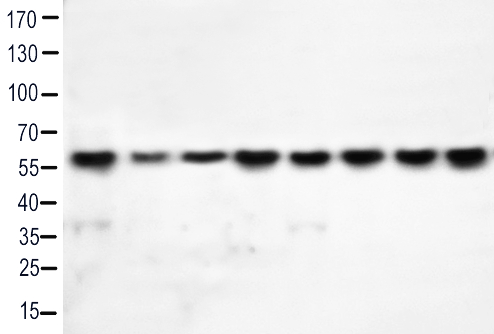


CYP27A1-Control


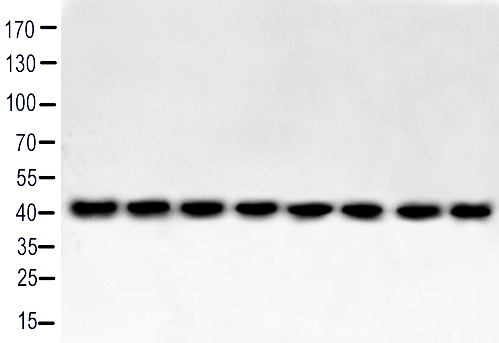


actin (CYP2R1-BA)


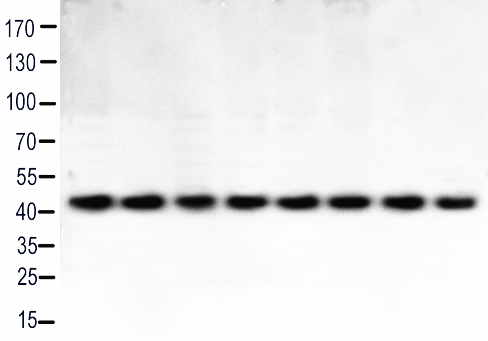


actin (CYP2R1-Control)


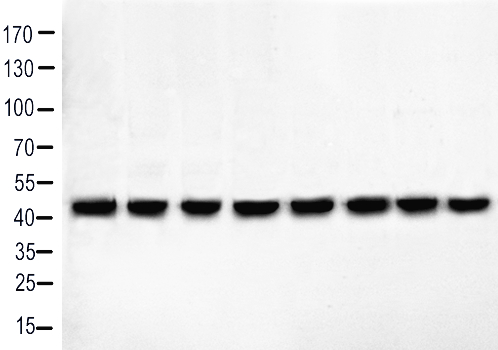


actin (CYP27A1-BA)


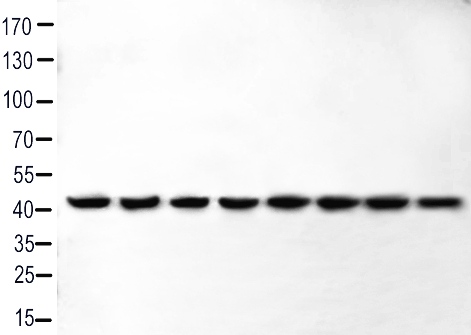


actin (CYP27A1-Control)

**Supplemental images for Figure 5**


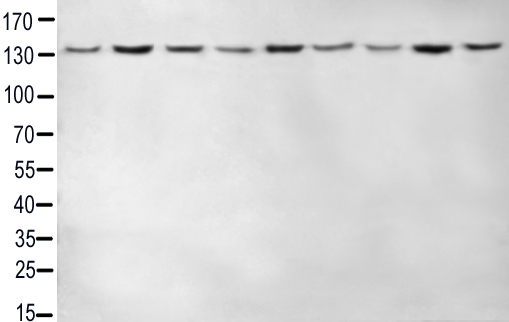


Col-I


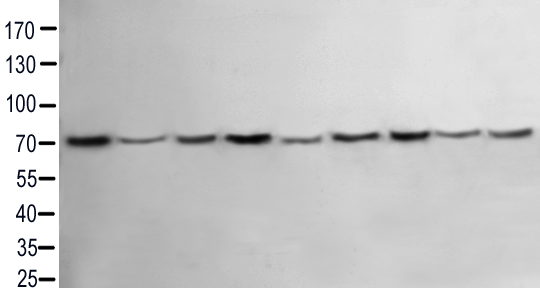


MMP2


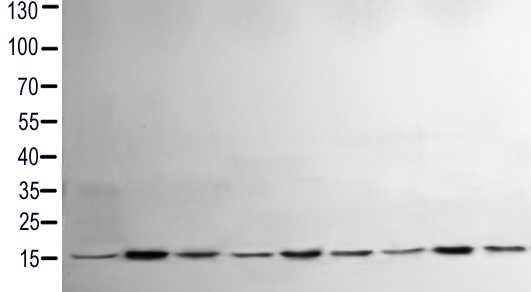


TGF-β1


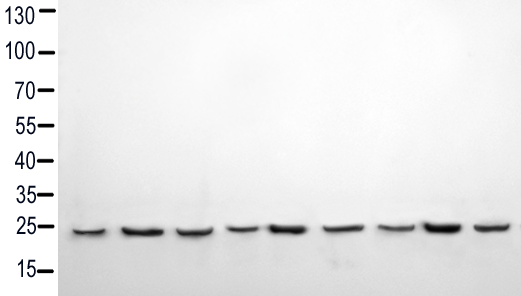


TIMP-1


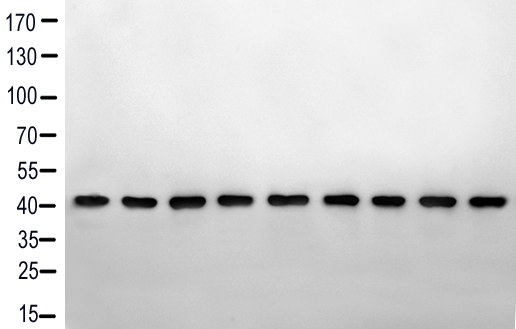


Actin

**Supplemental images for Figure 6**


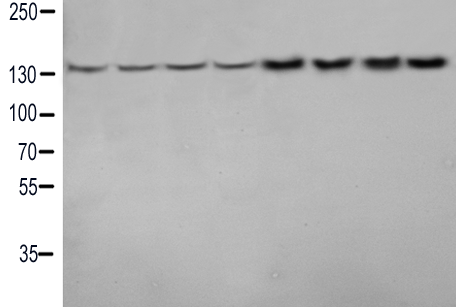


Col-1-1


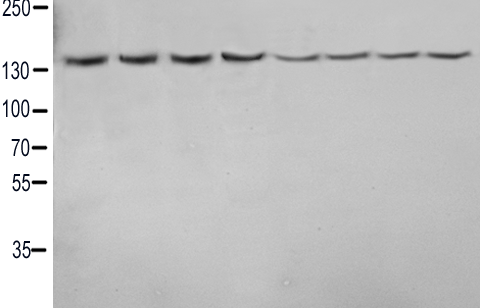


Col-1-2


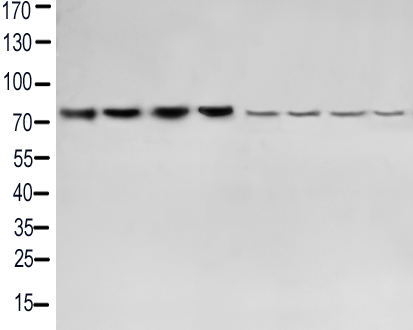


MMP2-1


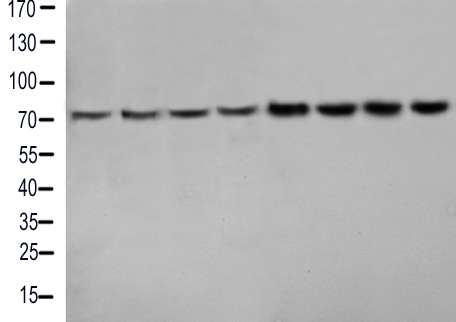


MMP2-2


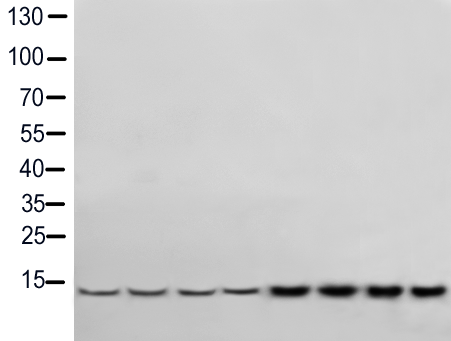


TGF-β1-1


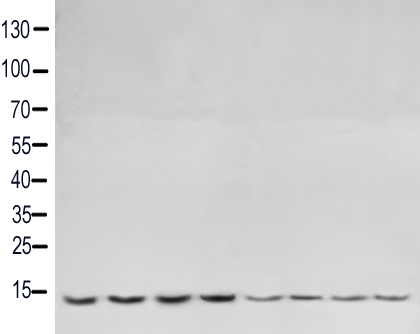


TGF-β1-2


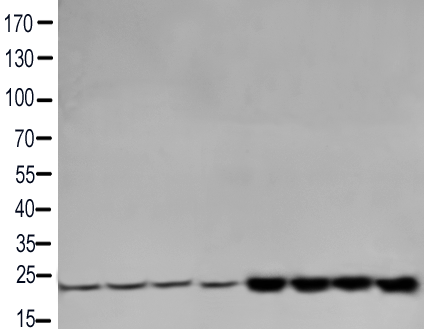


TIMP1-1


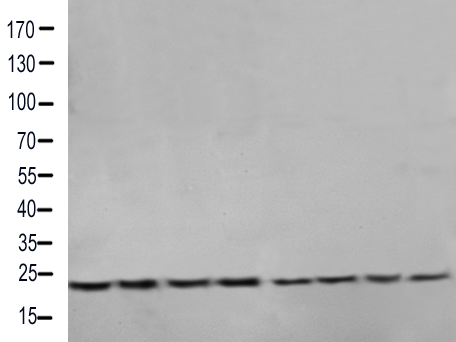


TIMP1-2


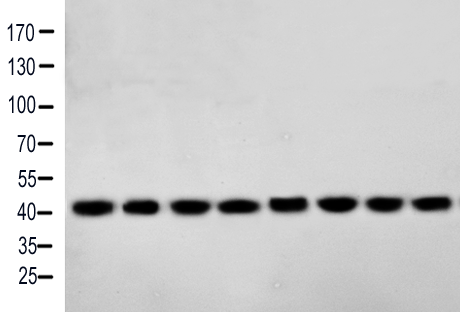


Actin-1


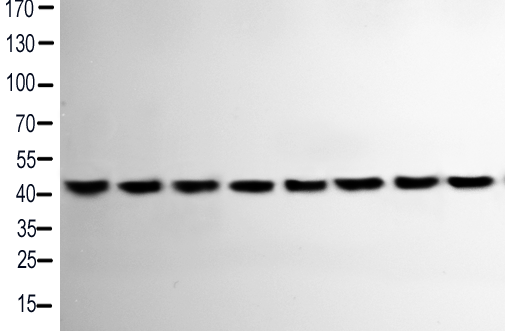


Actin-2
